# Topographic Mapping of Isolated Thalamic Infarcts Using Vascular and Novel Probabilistic Functional Thalamic Landmarks

**DOI:** 10.1007/s00062-022-01225-3

**Published:** 2022-11-22

**Authors:** Maximilian Rauch, Jan-Rüdiger Schüre, Franziska Lieschke, Fee Keil, Eike Steidl, Se-jong You, Christian Foerch, Elke Hattingen, Stefan Weidauer, Martin A. Schaller-Paule

**Affiliations:** 1 Institute for Neuroradiology, University Hospital, Johann Wolfgang Goethe-University, Theodor Stern Kai 7, 60590 Frankfurt am Main, Germany; 2 Department of Neurology, University Hospital, Johann Wolfgang Goethe-University, Theodor Stern Kai 7, 60590 Frankfurt am Main, Germany; 3 Department of Psychiatry and Psychotherapy, University Medical Center of the Johannes Gutenberg-University, Untere Zahlbacher Str. 8, 55131 Mainz, Germany

**Keywords:** Ischemic stroke, Cerebral infarction, Thalamic nuclei, Magnetic resonance imaging, Diffusion magnetic resonance imaging, Brain mapping

## Abstract

**Purpose:**

We aimed to re-evaluate the relationship between thalamic infarct (TI) localization and clinical symptoms using a vascular (VTM) and a novel functional territorial thalamic map (FTM).

**Methods:**

Magnetic resonance imaging (MRI) and clinical data of 65 patients with isolated TI were evaluated (female *n* = 23, male *n* = 42, right *n* = 23, left *n* = 42). A VTM depicted the known seven thalamic vascular territories (VT: inferolateral, anterolateral, inferomedial, posterior, central, anteromedian, posterolateral). An FTM was generated from a probabilistic thalamic nuclei atlas to determine six functionally defined territories (FT: anterior: memory/emotions; ventral: motor/somatosensory/language; medial: behavior/emotions/nociception, oculomotor; intralaminar: arousal/pain; lateral: visuospatial/somatosensory/conceptual and analytic thinking; posterior: audiovisual/somatosensory). Four neuroradiologists independently assigned diffusion-weighted imaging (DWI) lesions to the territories mapped by the VTM and FTM. Findings were correlated with clinical features.

**Results:**

The most frequent symptom was a hemisensory syndrome (58%), which was not specific for any territory. A co-occurrence of hemisensory syndrome and hemiparesis had positive predictive values (PPV) of 76% and 82% for the involvement of the inferolateral VT and ventral FT, respectively. Thalamic aphasia had a PPV of 63% each for involvement of the anterolateral VT and ventral FT. Neglect was associated with involvement of the inferolateral VT/ventral FT. Interrater reliability for the assignment of DWI lesions to the VTM was fair (κ = 0.36), but good (κ = 0.73) for the FTM.

**Conclusion:**

The FTM revealed a greater reproducibility for the topographical assignment of TI than the VTM. Sensorimotor hemiparesis and neglect are predictive for a TI in the inferolateral VT/ventral FT. The hemisensory syndrome alone does not allow any topographical assignment.

## Introduction

Thalamic infarcts (TI) account for approximately 3.1–4.4% of all ischemic strokes and despite their small size they are often associated with major neurological deficits [1–3]. Due to its strategic localization, the thalamus is deeply embedded in functional brain connectivity and plays a key mediating role in motor, sensory, coordinative, memory, cognitive and behavioral functions [1, 4–7]. The clinical diagnosis of TI may be challenging, since patients may present with a wide variety of symptoms depending on infarct location, volume and lateralization [1, 7–9].

However, the reported symptoms and syndromes associated with the involvement of certain vascular thalamic territories (VT: anterolateral—tuberothalamic artery; inferomedial—paramedian arteries; inferolateral—thalamogeniculate artery; posterior—posterior medial and lateral choroidal arteries; see Table 1) are not consistent in the literature [1, 6–8, 10]. Magnetic resonance imaging (MRI), in particular diffusion-weighted imaging (DWI), may provide valuable information on the topographic allocation of TI, as location and extent of infarction guide further diagnostic work-up and treatment and could have impact on prognosis [1, 3, 4, 11]. Previous studies investigating the relationship between infarct location and symptoms mostly relied on computed tomography (CT) alone [1, 12], were based on both CT and MRI [4, 10, 13] or focused on specific aspects of TI [5, 14–16]. The aim of our study was to (re-)evaluate the attribution of symptoms with respect to vascular thalamic territories. Furthermore, we aimed to create a novel territorial model based on functional thalamic properties.

**Table 1:** Thalamus-supplying vessels, synonyms and associated vascular syndromes [7, 8]

Territory	Supplying artery	Synonyms	Neurological symptoms
Anterolateral	Tuberothalamic artery	Anterior thalamoperforating artery Polar artery Inferior thalamic peduncle Anterior thalamosubthalamic paramedian artery Premamillary branch of thalamotuberian pedicle	Amnestic syndromes Memory disorders Apathy Disorientation Behavior abnormalities Aphasic syndromes Personality changes
Inferomedial	Paramedian arteries	Posterior thalamoperforating arteries Thalamoperforating pedicle Deep interpeduncular artery Retromamillary pedicle Posterior thalamosubthalamic artery	Disturbance of vigilance Transcortical aphasia Amnestic disorders up to thalamic dementia Psycho-organic symptoms Vertical gaze palsy
Inferolateral	Thalamogeniculate artery	Thalamogeniculate pedicle Inferolateral branches	Hemiparesis Hemiataxia Hemisensory syndromes Thalamic pain syndrome (Dejerine-Roussy syndrome) Vegetative disorders
Posterior	Posterior medial and lateral choroidal arteries	Posterior choroidal artery branches: medial and lateral branches	Visual field deficits/loss Hemisensory syndromes Dystonia Tremor

## Material and Methods

### Data Acquisition

Ethics approval for this retrospective study was obtained from the local authority. We reviewed our institutional radiological database for stroke patients who underwent MRI from 2010 to 2020, scanning a total of 6289 patients with ischemic stroke that presented to our stroke unit. Search terms applied in this collective included “MRI” and “thalamic stroke”, “thalamic infarction” or “infarction of the thalamus”. Only cases with both clinically and radiologically confirmed diagnosis of TI were included (*n* = 165). Patients with additional acute ischemia outside the thalamus (*n* = 47), or with underlying basilar artery occlusion (*n* = 48) as well as patients with bilateral TI (*n* = 5) were excluded. Data of the remaining 65 patients with isolated TI were used for further analysis.

### Imaging

MRI examinations were performed on two scanners, and the undermentioned sequences were used for further analysis. Philips Achieva 1.5 T (Philips Health Systems, Eindhoven, The Netherlands):

- T1 weighted, time repetition (TR) 3.8 ms, time echo (TE) 1.7 ms, flip angle 8°, section thickness 2.2 mm, matrix 320 × 320, field of view (FOV) 350 mm^2^
- Diffusion weighted imaging (DWI) axial, TR 3240 ms, time echo 75 ms, slice thickness 5 mm, gap 0.5 mm, matrix 256 × 256, FOV 220 mm^2^

Siemens Skyra 3.0T (Siemens Healthineers, Erlangen, Germany):

- T1 weighted, TR 3.1 ms, TE 1.4 ms, flip angle 8°, section thickness 2.2 mm, matrix 320 × 320, FOV 350 mm^2^
- DWI axial, TR 3800 ms, TE 95 ms, slice thickness 5 mm, gap 0.5 mm, matrix 384 × 384, FOV 230 mm^2^

### Thalamic Maps

**Fig. 1:**
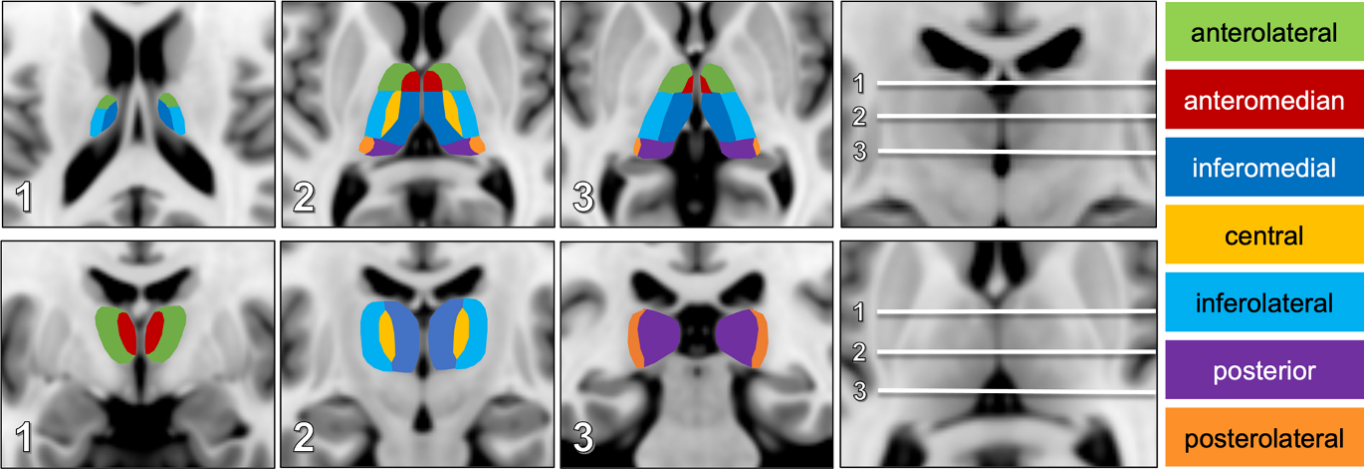
Vascular thalamic map: sectional topography of vascular thalamic territories in axial (*upper row*) and coronal sections (*lower row*) projected onto the MNI-152 standard template

A vascular thalamic map (VTM) including the four traditional and three variant type VTs as described by Carrera et al. [10] was manually created based on literature ([8, 10, 12, 17]; Fig. 1) and projected onto the Montreal Neurosciences Institutes (MNI) 152 standard template with 1 mm isotropic resolution [18].

For creation of the VTM, we used the four traditional vascular thalamic territories (see Table 1) and, in addition, variant territories based on variable arterial supply and border zone ischemia [10]:

1. The anterolateral territory—supplied by the tuberothalamic artery that originates from the posterior communicating artery.
2. The inferomedial territory—supplied by the paramedian arteries arising from the pre-communicating (P1) segment of the posterior cerebral artery (PCA).
3. The inferolateral territory—supplied by the thalamogeniculate artery that arises from the post-communicating P2 segment of the PCA.
4. The posterior territory—supplied by the posterior medial and lateral choroidal arteries that usually originate from the post-communicating P2-segment of the PCA.
5. The central territory—defined by the central thalamic part comprising parts of adjacent vascular territories.
6. The anteromedian territory—including the anterior part of the paramedian territory and the posterior part of the anterior territory.
7. The posterolateral territory—defined by infarcts involving the classical inferolateral and posterior territories, combining the posterior part of the inferolateral territory and the anterior part of the posterior territory.

**Fig. 2:**
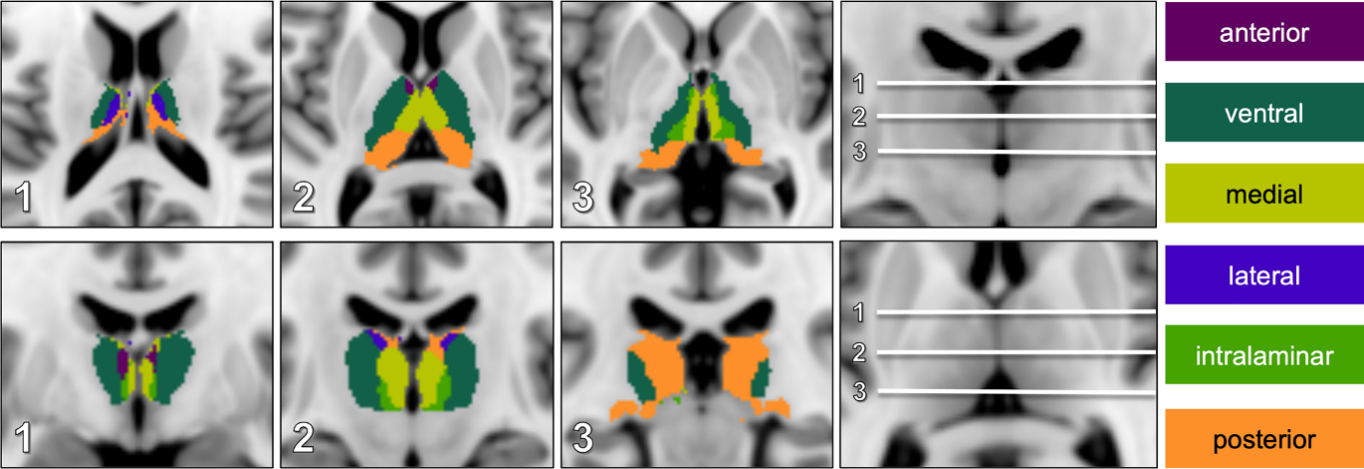
Functional thalamic map: sectional topography of functionally defined thalamic territories in axial (*upper row*) and coronal sections (*lower row*) projected onto the MNI-152 standard template

Using the open source FreeSurfer software suite (https://surfer.nmr.mgh.harvard.edu, RRID:SCR_001847), a novel thalamic map including six functionally defined territories (FTM; Fig. 2) was generated from a freely available probabilistic thalamic nuclei atlas by Iglesias et al. [19]. This atlas was created by using ex vivo MRI and histology and consists of 26 thalamic subregions that are based on the topography of the thalamic nuclei.

To ensure an accurate correlation of neurological symptoms and functional territory (FT), the 26 subregions were grouped into 6 territories, considering both the topography and the following fundamental thalamic functions:

- anterior: memory, emotions
- ventral: motor, somatosensory, language (left)
- medial: behavior, emotions, nociception, oculomotor
- intralaminar: arousal, pain
- lateral: visuospatial, somatosensory, conceptual/analytic thinking
- posterior: audiovisual, somatosensory

Territorial maps were then transferred from FreeSurfer space onto the MNI-152 standard template using MATLAB (MathWorks, Natick, MA, USA; version R2013b; RRID:SCR_001622) and converted to the Neuroimaging Informatics Technology Initiative (nifti) file format. In the next step, subregions were selected and combined according to the six predefined territories. The functional thalamic map (Fig. 2) was created to facilitate evaluation of MRIs.

### Data Analysis

MRIs were reviewed by four experienced neuroradiologists and diffusion-weighted imaging (DWI) lesion location was assessed at the same time using templates of both the VTM and FTM (Fig. 1 and 2). The raters were aware of the patients’ clinical symptoms.

If more than one territory appeared to be affected, the raters were required to opt for the territory that was predominantly affected by the DWI lesions. For discrepancies, final locations of lesions were determined in consensus with a fifth senior neuroradiologist.

Clinical data included baseline demographic characteristics, type and severity of symptoms as assessed by a neurological examination and NIHSS score at admission. Data were then analyzed by two neurologists and correlated with imaging findings.

To generate lesion overlay maps, DWI (b = 1000 s/mm^2^) images were aligned via co-registered three-dimensional T1-weighted data onto the MNI-152 standard space template. For this purpose, T1-weighted data were brain extracted and tissues segmented using the software tools BET and FAST from the FMRIB software library (FSL, version 5.0.7, https://surfer.nmr.mgh.harvard.edu, RRID:SCR_001847) toolbox. DWI (b = 0 s/mm^2^) images were aligned with the T1-weighted dataset via a boundary-based registration according to the segmented white matter. The T1-weighted data set was aligned to the MNI-152 template using a combination of linear and nonlinear registration. By combining the first (DWI to T1-weighted) and second (T1-weighted to MNI-152) transformation matrices, the co-registration was then applied on the DWI (b = 1000 s/mm^2^) images. TI masks were manually marked based on DWI in MNI-152 standard space and an overlap was used to generate corresponding lesion overlay maps.

### Statistical Analysis

Statistical analysis was performed using Microsoft Excel version 16.49 (Microsoft, Redmond, WA, USA) and GraphPad Prism version 6 (GraphPad Software, San Diego, CA, USA). Values are reported as the mean and standard deviation unless otherwise specified. Interrater agreement was evaluated using Fleiss’ kappa. Normal data distribution was ascertained using the D’Agostino-Pearson omnibus normality test.

## Results

A total of 65 patients (female *n* = 23, male *n* = 42) with isolated TI were included in the analysis. Mean age was 64 years (range 10–93 years). The majority of 42 patients (66.7%) presented with left sided TI, whereas only 23 patients (33.3%) had right sided TI. Clinical findings with respect to VTs and FTs are shown in Tables 2 and 3, respectively.

**Table 2:** TI characteristics regarding vascular territories

Vascular territory		Inferolateral	Anterolateral	Inferomedial	Posterior	Central	Anteromedian	Posterolateral
Supplying vessel^a^		Thalamogeniculate a	Tuberothalamic a	Paramedian aa	Posterior choroidal aa	Border zone variant	Border zone/variant	Border zone/variant
–		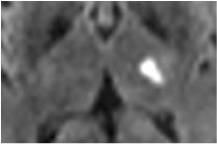	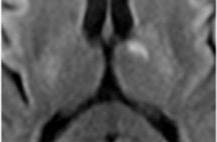	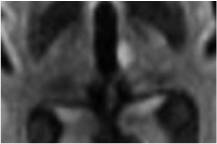	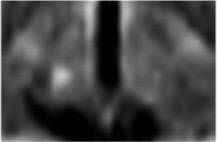	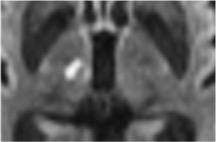	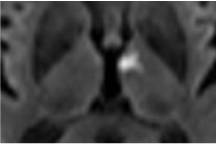	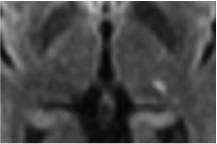
*n*		26	10	10	1	12	1	5
Male/female		17/9	6/4	5/5	0/1	10/2	1/0	3/2
Age (years, mean)		67	72	60	50	64	58	43
Left/right		15/11	7/3	8/2	1/0	7/5	0/1	4/1
NIHSS on admission (median; IQR)		4 (2–5)	4 (1.5–6.5)	4 (1.5–6.5)	2	1 (1–2)	2	2 (1–4.5)
DWI lesion volume (mm^3^) (median, IQR)		634 (307–956)	1011 (702–1811)	881 (503–1333)	207	629 (246–1344)	392	740 (377–1907)
Leading symptom (s)		Hemiparesis, hemisensory disturbance	Hemiparesis, thalamic aphasia, dysarthria	Ambiguous	Indeterminate	Hemisensory disturbance	Indeterminate	Hemisensory disturbance
Hemiparesis	*n*	16 (62%)	6 (60%)	4 (40%)	1	1 (8%)	1	1 (20%)
Hemisensory disturbance		19 (73%)	2 (20%)	2 (20%)	1	9 (75%)	–	5 (100%)
Ataxia		6 (23%)	–	2 (20%)	1	–	–	1 (20%)
Thalamic aphasia		1 (4%)	5 (50%)	1 (10%)	–	1 (10%)	–	–
Dysarthria		8 (31%)	5 (50%)	2 (20%)	–	2 (17%)	–	–
Facial paresis		5 (19%)	3 (30%)	2 (20%)	–	–	–	–
Neuropsychological deficits		2 (8%)	2 (20%)	2 (20%)	–	–	1	–
Decreased vigilance		1 (4%)	2 (20%)	1 (10%)	–	–	–	–
Amnesia		–	2 (20%)	2 (20%)	–	–	–	–
Oculomotor disturbance		–	1 (10%)	1 (10%)	–	–	–	–
Vertigo/gait		1 (4%)	1 (10%)	–	–	2 (17%)	–	1 (20%)
Thalamic pain		1 (4%)	1 (10%)	–	–	–	–	–
Behavioral changes		–	–	1 (10%)	–	–	1	–
Dysphagia		2 (8%)	–	–	–	–	–	–
Neglect		2 (8%)	–	–	–	–	–	–
Annotations	–	Co-occurrence of hemisensory disturbance and hemiparesis had a PPV of 76% for involvement of this VT	Occurrence of thalamic aphasia had a PPV of 63% for involvement of this VT	Patients presented with widespread neurological symptoms, no leading symptom identified	Patient did not have involvement of the geniculate bodies	–	–	All patients presented with hemisensory disturbance
		Only VT in which neglect and dysphagia occurred as symptoms in TI involvement						None of the patients had involvement of the medial and lateral geniculate bodies

*IQR* interquartile range, *DWI* diffusion weighted imaging, *NIHSS* National Institutes of Health Stroke Scale, *PPV* positive predictive value, *TI* thalamic infarction, *VT* vascular territory

^a^For synonyms see Table 1

**Table 3:** TI characteristics regarding functional territories

Functional territory		Anterior	Ventral	Medial	Intralaminar	Lateral	Posterior
–		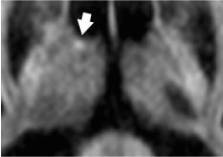	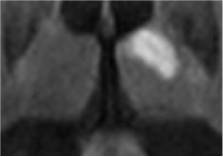	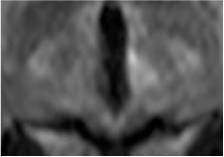	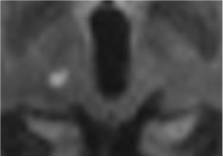	–	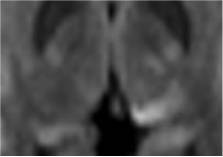
*n*		2	41	13	5	None	4
Male/female		1/1	28/13	8/5	4/1	–	1/3
Age (years, mean)		65; 89	66	62	62	–	43
Left/right		1/1	29/12	9/4	2/3	–	1/3
NIHSS on admission (median; IQR)		4; 6	3 (1.4–4.5)	2 (1–7)	2 (1–4)	–	1 (1–1.75)
DWI lesion volume (mm^3^) (median, IQR)		15; 628	871 (585–1361)	769 (323–1166)	471 (293–701)	–	215 (139–454)
Leading symptom(s)		Indeterminate	Hemisensory disturbance, hemiparesis	Hemiparesis, dysarthria	Hemisensory disturbance	–	Hemisensory disturbance
Hemiparesis	*n*	1 (50%)	23 (56%)	5 (38%)	1 (20%)	–	–
Hemisensory disturbance		–	25 (61%)	4 (31%)	5 (100%)	–	4 (100%)
Ataxia		–	8 (20%)	1 (8%)	–	–	1 (25%)
Thalamic aphasia		1 (50%)	5 (12%)	2 (15%)	–	–	–
Dysarthria		1 (50%)	11 (27%)	5 (38%)	–	–	–
Facial paresis		1 (50%)	5 (12%)	2 (15%)	2 (40%)	–	–
Neuropsychological deficits		1 (50%)	2 (5%)	3 (23%)	1 (20%)	–	–
Decreased vigilance		–	1 (2%)	3 (23%)	–	–	–
Amnesia		1 (50%)	1 (2%)	2 (15%)	–	–	–
Oculomotor disturbance		–	1 (2%)	1 (8%)	–	–	–
Vertigo/gait		–	3 (7%)	1 (8%)	–	–	1 (25%)
Thalamic pain		1 (50%)	–	–	1 (20%)	–	–
Behavioral changes		–	–	2 (15%)	–	–	–
Dysphagia		–	2 (5%)	–	–	–	–
Neglect		–	2 (5%)	–	–	–	–
Annotations	–	Patients presented with a wide range of symptoms, no leading symptom elaborated	Most frequently affected FT	Affection predominantly resulted in motor symptoms	None of the patients showed decreased vigilance	–	–
			PPVs for involvement of this FT: Hemiparesis + hemisensory loss: 82% Thalamic aphasia: 63% Dysarthria: 64%				

*IQR* interquartile range, *PPV* positive predictive value, *FT* functional territory

Interrater reliability for the neuroradiological assignment of infarcts to the VTM was fair (κ = 0.36), but good (κ = 0.73) for the FTM. Consistency between all raters was best for the anterolateral VT (6/10; 60%) and for the ventral FT (31/41; 75.6%). The lowest interrater agreement was found for the central VT (2/12; 16.2%) and for the intralaminar FT (2/5; 50%). The most frequently affected territories were the inferolateral VT (26/65; 40%) and the ventral FT (41/65; 63.1%).

**Fig. 3:**
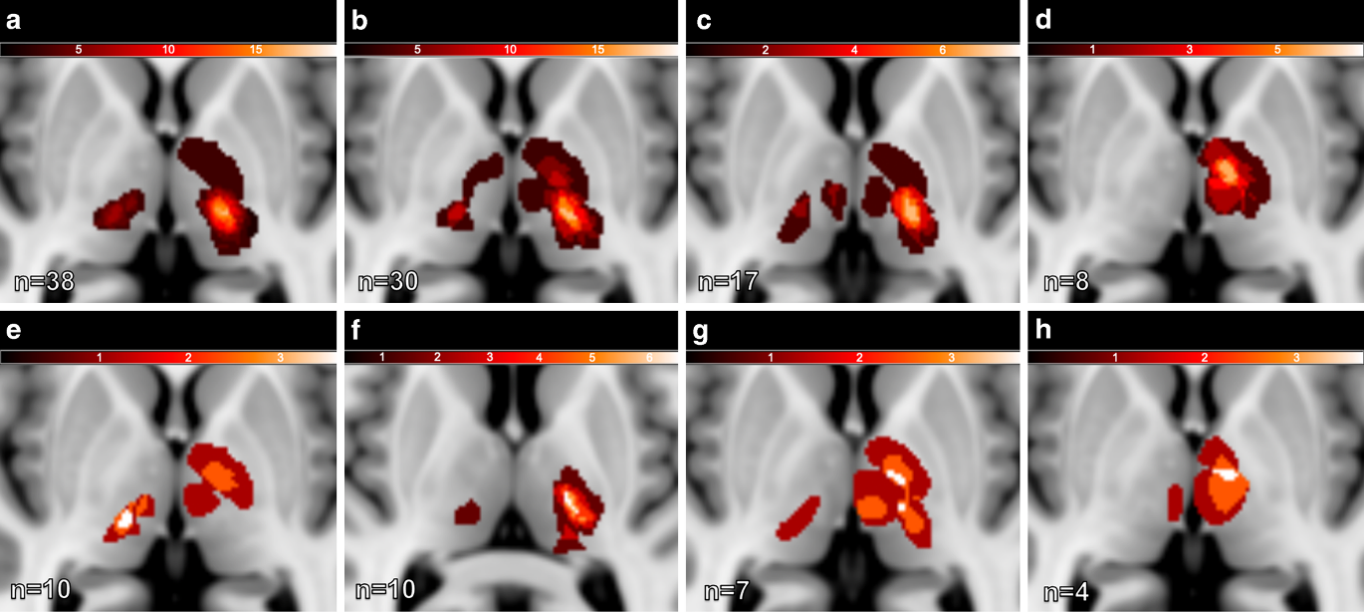
Thalamic infarction lesion overlay maps in axial orientation concerning most frequently observed symptoms. The overlay images are projected onto the MNI-152 space. **a** Hemisensory, **b** hemiparesis, **c** dysarthria, **d** aphasia, **e** facial paresis, **f** ataxia, **g** neuropsychological, **h** vigilance

Evaluation of lesion overlay maps (Fig. 3) showed an approximate geometrical congruence of affected areas for motor symptoms (hemiparesis, facial paresis, dysarthria). All patients with aphasia (*n* = 8) had TI located in the rostral portions of the left thalamus (anterolateral VT, inferomedial VT, central VT, inferomedial VT) (Fig. 3). Patients with ataxia had lesion location in the lateral portions of the thalamus in which the inferolateral VT was affected in 60%.

Based on the overlays, concordance between actual clinical symptoms and those expected based on the anatomic location of TI was found in 46/65 patients (70.1%) when applying the VTM and in 46/65 (78.5%) patients when applying the FTM. Concordance between the manually assigned TI localizations (as per consensus) and the corresponding locations resulting from the overlays was 62/65 (95.4%) for the VTM and 63/65 (97.0%) for the FTM.

## Discussion

We evaluated the topographic mapping of stroke symptoms in patients with isolated TI using two different territorial classifications of the thalamus: (1) the common separation based on the vascular supply (VTM) and a (2) functional subdivision of the thalamus based on a functional probabilistic map (FTM). We depicted the acute TI on DWI and four radiologists performed the topographic mapping based on the VTM and FTM. The interrater reliability in mapping DWI lesions to the VTM was rather low. Clinical symptoms were known to the raters, therefore, this knowledge might have influenced the choice of VTs. Due to the variability of VTs, defined clinical symptoms and syndromes sometimes cannot be clearly assigned to a particular VT, resulting in dichotomous allocation. The inconsistency of VTs may be based on thalamus-supplying vascular variants, which are common [20–24], but not considered in the traditional vascular-based approach. Consecutively, our study showed a better interrater reliability when using the FTM compared to the VTM.

Moreover, from a neuroradiological point of view, thalamus-supplying vessels are of such a small caliber that they usually are not seen in clinical routine MR or CT angiography. Therefore, attribution of TI to a particular vessel occlusion and a single VT is often not feasible [7, 14].

In contrast, a specific clinical symptomatology can usually be functionally anatomically well assigned to thalamic nuclei groups. We therefore created a new territorial map based on functional thalamic properties. There was a good interrater agreement in mapping infarcts on DWI (b = 1000 s/mm^2^) to this newly created FTM. Only the intralaminar FT (corresponding to the central VT) showed a low interrater agreement most likely due to the smallest volume of all thalamic territories.

Therefore, the mapping of stroke symptoms in isolated TI seems to be more valid using the FTM. This was to be expected, since here the topographical assignment of the thalamic regions is based on their respective function. Conversely, however, our results show that these classifications, which are mostly based on empiricism, seem to be valid because the assignment was successful. Nevertheless, this encompasses not all neurological symptoms. The topographic mapping for the sensorimotor hemiparesis and the rare neglect was reliable, while the sensory hemisyndromes and aphasia could not be assigned with certainty even on the FTM. Hemisensory disturbance occurred as a predominant symptom in 57% of patients without a preponderance for a certain territory. Regarding its assignment to territories, it is a non-specific symptom and occurred as the most frequent symptom in four of seven VTs and three of the six FTs.

However, a study investigating pure sensory syndromes in TI showed that sensory dysfunction and disturbance were more common in TI involving the ventrocaudal nucleus and the ventro-oral nucleus intermedius [16]. These nuclei are part of the ventral FT, which was most commonly affected in our patient group, accordingly.

Moreover, the co-occurrence of hemisensory disturbance and hemiparesis showed a positive predictive value (PPV) of 76% and 82% for the involvement of the inferolateral VT and ventral FT, respectively. The appearance of thalamic aphasia had a PPV of 63% each for involvement for the anterolateral VT and the ventral FT, nevertheless, no assignment to a specific territory was possible. All eight patients with aphasia had TI located on the left side affecting the rostral portions of the thalamus, which is consistent with recent reports suggesting that appropriate TIs disrupt input from left cortical areas to anterior thalamic nuclei, leading to aphasic symptoms [5]. Ataxia was predominantly associated with involvement of the lateral portions of the thalamus, including the inferolateral VT in 60%, and the ventral FT in 80%. The finding may be explained by affection of the ventral lateral nucleus (VL) that is located in these territories and forms part of the motor functional division [12, 25]. Dysarthria was predominantly associated with involvement of the inferolateral VT and ventral FT, with a PPV of 64% for the involvement of the ventral FT.

As mentioned above, interrater agreement was lowest for the small central VT, as its location may impede a distinct delineation from adjacent territories. Carrera et al. found a wide variety of neurological and neuropsychological syndromes as a result of involvement of adjacent thalamic structures [10]. They determined hypesthesia in all of these patients as the leading symptom. Although the involvement of the central VT occurred more often in our patients (18.5%), the main symptom of hemisensory disturbance likewise occurred in 75% of patients.

However, the assignment of stroke symptoms to the vascular supply of the thalamus may be more relevant than functional mapping because it does not answer the most important questions in acute stroke, whether it is caused by microangiopathy or macroangiopathy or whether there is an embolic source in the anterior or posterior circulation. Anyhow, nowadays this should be answered by CT angiography or MR angiography performed in the context of stroke evaluation. Moreover, the overlap of the thalamic blood supply of the different territories is too pronounced to be adequately defined.

Previous studies pointed out that involvement of particular vascular territories was not significantly associated with a specific cause for infarction, although it was hypothesized that affection of the inferolateral vascular territory results from small vessel hypertensive arteriolopathy in the thalamogeniculate artery [17]. In contrast, cardioembolism was found more often in infarction of the inferomedial territory which is supplied by the paramedian arteries [1, 7, 15, 26]. Therefore, the information on stroke etiology given by TI localization based on a vascular map may be limited. Our results underline this overlap of symptoms and blood supply, so that functional thalamic mapping should be more appropriate for clinical use. A potential criticism of our study is the fact that we assigned the infarcts on DWI (b = 1000 s/mm^2^) to a single territory for the purpose of further analysis. In lesions overlapping two or more territories, raters were instructed to define the majorly affected territory. However, a recent study showed that in isolated TI, 97% of ischemic lesions were confined to one vascular territory, whereas in posterior cerebral artery territory infarct, top of the basilar artery syndrome and extended posterior circulation infarctions, ischemic lesions tended to involve multiple thalamic territories [17].

Another criticism may be that our novel functional thalamic map includes six vascular territories in contrast to the seven traditional vascular territories typically mentioned in literature [7, 8]. However, our intention was to establish a straightforward model with high discriminatory power that represents basic thalamic functions.

In conclusion, the presented results underline that the topographic mapping of stroke symptoms in patients with isolated TI based on vascular territories is often not possible. Therefore, we propose a new more reliable classification system for TIs based on functional anatomy that demonstrated a better reproducibility compared to the traditional vascular territory model.

In daily clinical practice, radiological description of TI using the traditional vascular territory model may leave stroke physicians in uncertainty of the affected anatomy and expected associated clinical syndrome. The possible benefit of specifying the occluded (perforator) artery often bears no immediate clinical relevance and may remain of merely academic value. In contrast, a functional territory model enables the treating neurologist to link TI lesions more reliably to the observed clinical findings. When the affected functional territories satisfactorily match the observed neurological deficits, therapeutic and rehabilitative measures may be arranged promptly. However, if the respective TI lesion is not fully explanatory for the observed clinical syndrome (e.g., impaired consciousness, personality changes, aphasia), clinicians can be confidently encouraged to initiate further diagnostic work-up, such as additional cerebrospinal fluid analysis, electroencephalography, or laboratory assessment. In summary, the proposed clinical radiologic model may help to more consistently assign TI to specific neurological symptoms, and therefore improve the interaction between radiologists and stroke physicians towards a more focused patient care.

Effectiveness and user acceptance of the novel territorial model may be evaluated in follow-up studies using larger patient collectives. The new model may also be beneficial for further research investigating the role of thalamic lesions including correspondent research in movement disorders [27], dementia [28], neuropsychology or psychiatry [4, 29].
